# Genome-Wide Association Mapping for Seedling and Adult Plant Resistance to Stripe Rust in Synthetic Hexaploid Wheat

**DOI:** 10.1371/journal.pone.0105593

**Published:** 2014-08-25

**Authors:** Habtemariam Zegeye, Awais Rasheed, Farid Makdis, Ayele Badebo, Francis C. Ogbonnaya

**Affiliations:** 1 Ethiopian Institute of Agricultural Research, Addis Ababa, Ethiopia; 2 Crop Science Research Institute/National Wheat Improvement Centre, Chinese Academy of Agricultural Sciences (CAAS), Beijing, China; 3 Department of Plant Sciences, Quaid-i-Azam University, Islamabad, Pakistan; 4 Department of Field Crops, Faculty of Agriculture, University of Aleppo, Aleppo, Syria; 5 International Centre for Agricultural Research in the Dry Areas (ICARDA), Aleppo, Syria; 6 Research Program, Grains Research and Development Corporation, Barton, Australian Capital Territory, Canberra, Australia; USDA-ARS-SRRC, United States of America

## Abstract

Use of genetic diversity from related wild and domesticated species has made a significant contribution to improving wheat productivity. Synthetic hexaploid wheats (SHWs) exhibit natural genetic variation for resistance and/or tolerance to biotic and abiotic stresses. Stripe rust caused by (*Puccinia striiformis* f. sp. *tritici; Pst*), is an important disease of wheat worldwide. To characterise loci conferring resistance to stripe rust in SHWs, we conducted a genome-wide association study (GWAS) with a panel of 181 SHWs using the wheat 9K SNP iSelect array. The SHWs were evaluated for their response to the prevailing races of *Pst* at the seedling and adult plant stages, the latter in replicated field trials at two sites in Ethiopia in 2011. About 28% of the SHWs exhibited immunity at the seedling stage while 56% and 83% were resistant to *Pst* at the adult plant stage at Meraro and Arsi Robe, respectively. A total of 27 SNPs in nine genomic regions (1BS, 2AS, 2BL, 3BL, 3DL, 5A, 5BL, 6DS and 7A) were linked with resistance to *Pst* at the seedling stage, while 38 SNPs on 18 genomic regions were associated with resistance at the adult plant stage. Six genomic regions were commonly detected at both locations using a mixed linear model corrected for population structure, kinship relatedness and adjusted for false discovery rate (FDR). The loci on chromosome regions 1AS, 3DL, 6DS and 7AL appeared to be novel QTL; our results confirm that resynthesized wheat involving its progenitor species is a rich source of new stripe (yellow) rust resistance that may be useful in choosing SHWs and incorporating diverse yellow rust (YR) resistance loci into locally adapted wheat cultivars.

## Introduction

Stripe (yellow) rust (YR), caused by *Puccinia striiformis tritici* (*Pst*), is a major threat to wheat production in many areas. A recent experience is the stripe rust of near - epidemic proportions in the Central West Asia and North Africa (CWANA) and Sub-Saharan Africa regions in 2010 [1]. Previous YR epidemics have occurred in some major wheat producing regions including China, Europe, Australia, Ethiopia, South Africa, the US and South Asia [2]. In 2010, the breakdown in resistance conferred by *Yr27* and the absence of resistant cultivars in Ethiopia led to an expenditure of more than US$3.2 million on fungicides, and over 400 000 ha of wheat were estimated to have been infected by YR [1]. Similar epidemics were reported in Iran, Morocco, Syria and Turkey in the same year. Despite the concerted efforts to control the disease using fungicides, substantial losses were prevalent on highly susceptible cultivars in areas with high disease pressure. Breeding resistant cultivars is the most economical and environmentally best approach to reduce the use of fungicides and to reduce crop losses due to this disease.

To date, 54 YR resistance genes have been formally designated in wheat [3]. Most of these are race-specific, produce hypersensitive reactions, and interact with the pathogen in a gene-for-gene manner [4]. Such qualitative resistance is usually short-lived, owing to frequent changes in the pathogen population. Due to the rapid break down of commercially deployed resistance genes, characterization of diverse sources of resistance is continuously needed to replace the defeated genes. The alternate option is to deploy adult plant resistance (APR) genes conferring quantitative resistance, and in some cases APR genes are durable and confer resistance to multiple pathogens. Disease resistance can be transferred from cultivars and even from wild relatives of wheat by direct recombination or *via* bridge crosses or synthetic wheats [5].

Synthetic hexaploid wheat [(2n = 6x = 42, AABBDD), SHWs] obtained by the artificial crossing of durum wheat, *Triticum turgidum* L.ssp. *durum* (Desf.) Husn. (2n = 4x = 28, AABB) and *Aegilops tauschii* Coss. (2n = 2x = 14, DD) possess genetic diversity for resistance to several biotic (karnal bunt (*Tilletia indica* Mitra), cereal cyst nematode (*Heterodera avenae* Woll.), root knot nematode (*Meloidogyne naasi* Franklin), and green bug (*Schizaphis graminum* (Rondani)); and abiotic stresses as observed upon screening of the AABBDD SHWs [6], [7], [8], [9]. Resistance genes for leaf rust such as *Lr21, Lr22*, *Lr32, Lr39, Lr41* [10]; YR resistance gene *Yr28* [11] and Hessian fly resistance genes: *H13, H22, H23, H24* [12] were derived from *Ae. tauschii*. *Aegilops tauschii* is also known to have contributed three stem rust resistance genes: *Sr33, Sr45*, and *Sr46* which confer resistance to race Ug99 [13].

Recently, Rosewarne et al. [14] in a review article reported that about 140 quantitative trait loci (QTLs) have been identified in 49 chromosomal regions conferring resistance to YR. The QTLs were mapped using bi-parental populations that typically involve two or at most several parental accessions and, therefore, evaluated only a small fraction of the natural variation in a species [15]. QTL mapping and genome-wide association studies (GWAS) offer complementary approaches to understanding natural variation [16]. GWAS generally combine phenotype and genotype data from 100 or more accessions to identify loci with allele frequency correlations to phenotypic variation or environment [17]. This approach can thus incorporate a relatively large portion of natural variation in a species and localize associations to much smaller genomic regions, because the sampled diversity incorporates many more recombination events than traditional recombinant inbred lines and/or doubled haploid populations [18]. However, potential disadvantages of GWAS are the appearance of false-positives resulting from population structure, or associations that arise from multiple testing of thousands of single nucleotide polymorphism (SNPs) markers, as well as the potential to miss signal (false negatives) because of low power to detect small genetic effects and limitations due to allelic heterogeneity and non-additive effects among loci [18]. The recent development and availability of 9K SNP array in wheat which was used to characterize a wide array of hexaploid cultivars from many parts of the world is facilitating the use of SNPs in GWAS [19]. This SNP array have been used in genome wide association studies to identify genomic regions and/**or** markers for grain asparagine contents [20], resistance to Hessian fly [21], grain yield [22] and frost tolerance [23]. Association mapping thus offers the unique opportunity of linking diversity analysis, identification of marker-trait associations and molecular breeding.

This study was conducted to address the following three objectives: (1) to assess the diversity of disease resistance in SHWs to prevailing YR races in Ethiopia, (2) to carry out a genome wide search in SHWs for resistance loci to the prevailing YR races and identify SNP markers associated with such YR resistance which could be deployed in marker-assisted selection (MAS) and (3) to determine whether some SHWs contain yet un-characterized genes for YR resistance in wheat.

## Materials and Methods

### Genetic resources

A total of 192 genotypes were used for this study including 181 SHWs and eleven bread wheat cultivars (Kubsa, Galama, ET13A2, Meraro, Kenya Kudu, Dashen, Digelu, Enkoy, KBG-01, Simba and Morocco) as checks (Table S1). The check cultivars are known to show variable reaction to YR under field conditions in areas endemic for YR occurrence in Ethiopia. The SHWs were obtained from ICARDA, Aleppo – Syria and comprised of genotypes from CIMMYT, Mexico and Department of Primary Industries, Victoria, Australia. The check cultivars were obtained from Ethiopian Institute of Agricultural Research (EIAR), Kulumsa Experimental Station, Ethiopia.

### Phenotyping

#### Seedling stage

Five to six seeds of each SHW were grown in a 7 cm×7 cm×7 cm plastic pots. Each pot was filled with a potting mix which comprised of: compost, soil and sand at a ratio of 1∶1∶1 (v/v/v). When the first leaves were fully expanded, the seedlings were inoculated by spraying the most virulent Kubsa/Attila isolate urediospores suspended in light mineral oil (Soltrol 170) using an automizer. The Kubsa/Attila isolate is the most virulent and predominant isolate in the central highlands of Ethiopia. Inoculated plants were allowed to dry for 5 minutes and were fine-misted with water and placed in a wet plastic cage with a small amount of water at the bottom. The inoculated seedlings were incubated at 10°C for 24 hours in a dew chamber with relative humidity close to 100%. Seedlings were transferred to a greenhouse with mean temperature of about 18°C at the Ethiopian Institute of Agricultural research, Kulumsa experimental station. A week after inoculation, 2 grams of nitrogen fertilizer per 100 ml was added as liquid fertiliser to each pot. Disease assessment was carried out twice: on the 15^th^ and 18^th^ days after inoculation using 0–4 scale [24] based on the infection types. Low infection types (LITs = 0–2) were considered resistant, and infection type  = 2+ as intermediate while high infection types (HITs =  3–4) were rated susceptible. The check cultivars Dashen, Galama, Kenya Kudu, Kusba, Morocco and Wabe were susceptible, Enkoy, ETA 13-A2 and Simba were moderate and, Digelu and KBG-01 were resistance to YR at seedling stage.

#### Adult plant stage

Twenty seeds of each SHWs including the checks were planted in two rows of 20 cm spacing and 1 m length in two sites namely: Meraro (07°41′N 39°25′E) with an elevation of 3,030 meters and Arsi Robe (09°36′′′) with an elevation of 2435 meters above sea level, in Ethiopia in 2011. Six bread wheat check cultivars (Kubsa, Galama, ET 13-A2, K62954-4A, Meraro and Kenya Kudu) were planted within intervals of ten entries in the field that included the spreader row of the yellow rust susceptible cultivar Morocco. Kubsa and Galama are known to be susceptible, ET-13A2 as moderately susceptible and Meraro and Kenya Kudu are moderately resistant to resistant cultivars. Disease assessment started from the first incidence and continued at least four times at ten day intervals. Disease severity was assessed according to the modified Cobb's scale [25]. After the last disease score when the disease progress ceased, the field severity data was converted to Coefficient of Infection (CI) and the area under disease progress curve (AUDPC) was calculated following the method used by Wilcoxson et al. [26].

### DNA extraction and SNPs marker genotyping and Molecular analyses

Five seeds of each SHW were planted in 5 cm diameter pots filled with peat moss in a plastic house at ICARDA. Fresh leaf samples were collected from 17 day old seedlings and used for DNA extraction. DNA extraction was carried out according to Ogbonnaya et al. [27]. Subsequently, aliquots of DNA concentration of 50–100 ng/µl per sample were sent in a 96-well plate format to the Department of Primary Industries, Victoria – Australia for genotyping with high-density SNP markers, using the Wheat 9K SNP array [19].

#### Genetic diversity, PIC, MAF and genetic map

Genetic similarities between wheat lines were calculated with Dice coefficient based on the proportion of shared alleles using PowerMarker v. 3.0 [28]. Polymorphism information content (*PIC*) assessed the genetic diversity at each locus. *P* =  the number of polymorphic loci/the total number of loci. *PIC* was calculated as described by Anderson et al. [29]. *PIC* = 1-Σ*P*_ij_^2^, where the relative frequency of the *jth* allele for the *ith* locus was summed across all the alleles for the locus over all lines. The position of SNP markers along chromosomes in terms of genetic distance (cM) was based on the map reported by Cavanagh et al. [19], however, 111 markers remained unmapped. The integrated map of SNPs, SSRs, DArTs and genotyping by sequencing (GBS) markers [30] was used to identify physical position of SNPs associated with YR resistance.

#### Population structure

Population structure was estimated with 200 unlinked SNP markers using STRUCTURE software 2.3.3, which implements a model based Bayesian cluster analysis [31]. The number of subpopulations (K) was set from 2–20 based on admixture and correlated allele frequencies models. For each K, 10 runs were performed separately. Each run was carried out with 50 000 iteration and 500 000 burn-in period. A value of K was selected where the graph of *InPr* (X/K) peaked in the range of 2–20 sub-populations. For the selected K, 10 runs were performed again each with 100 000 iteration and 500 000 burn-in period.

#### Linkage disequilibrium (LD)

Genome-wide LD analysis was performed across A, B and D genomes for the complete association mapping set. LD was estimated as squared allele-frequency correlations (*r^2^*) between pairs of SNP markers according to Weir [32] using the GGT v 2.0 software. To depict the extent of LD between pairs of loci, *r^2^* values were plotted against inter-marker genetic distance (cM). Locally weighed polynomial regression (LOESS) curves were then fitted into the scatter plot using function ‘*smooth.spline*’ of R (R Development Core Team, 2011). Specifically, the 95th percentile in the distributions of *r^2^* of the selected loci was estimated as the threshold *r^2^* [33] on the assumption that LD was attributable to linkage. At its points of intersection with the LD decay curves, the threshold *r^2^* was plotted as a horizontal line in the LD scatterplot which provided estimates of the extent of LD. LD along chromosomes was assessed by a sliding window approach with 5 cM windows at 500 positions along the chromosomes.

#### Association analysis

Genomic regions associated with YR resistance were identified using the compressed mixed linear model (MLM) implemented in TASSEL v.3.0 [34]. A *P*-value was generated by fitting each SNP marker into the MLM that has the form, *y = Xβ+Qv+u+e*, where *y* is the vector of the phenotypic values (BLUPs), *X* is the vector of SNP marker genotypes, *β* is the vector of marker fixed effects to be estimated, *Q* is population structure matrix (derived from Structure analysis), *v* is a vector of fixed effects due to population structure, *u* is the vector of random effects and *e* is the vector of residuals. The variance of *u* is derived as, Var (*u*) = 2KV*g*, where K represents the relative kinship matrix inferred from genotypes based on the proportion of shared alleles and V*g* is the genetic variance. The variance of *e* is derived as Var (*e*) = V*R*, where V*R* is the residual variance.

The critical *P*-values for assessing the significance of marker-trait associations in the MLM were calculated based on positive false discovery rate (pFDR; Q values), a multiple test correction method proposed by Storey [35]. These FDR-adjusted *P*-values were found to be highly stringent, probably owing to the use of all markers as independent tests in the correction. Considering the likelihood of over adjustment of the *P*-values owing to the mutual dependency of SNP in LD [36], and the potential risk of type II error, a more liberal criterion was used to determine threshold *P*-values for marker-trait association. We followed the approach of Pasam et al. [37] who suggested that the bottom 0.1 percentile of the distribution of *P*-values obtained can be considered as significant. As a consequence, a threshold *P*-values of ≤0.005 or ≤0.05 which roughly corresponds to the bottom 0.1 percentile in the present GWAS was used to declare significant QTL for Yr resistance. To provide a complimentary summary of declared putative QTL, Manhattan plots were generated using a script written in R (R Development Core Team, 2011). Significant markers were also inspected for correspondence with genomic regions known to harbour QTL for *Yr* resistance genes based on consensus maps in the CMap database (http://ccg.murdoch.edu.au/cmap/ccg-live/) and those reported in Rosewarne et al. [14].

## Results

### Phenotypic variations for stripe rust resistance in SHWs

**Figure 1:**
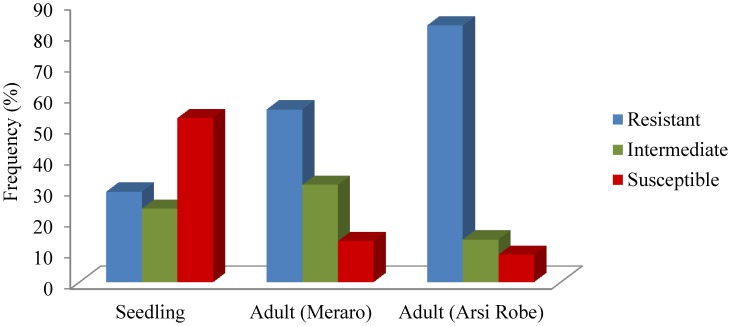
Frequency distribution of response to stripe rust in synthetic hexaploid wheat and bread wheat cultivars.

Reaction to YR in SHWs at the seedling and adult plant stages at Meraro and Arsi Robe Ethiopia are summarized (Figure 1, Table S4). Continuous variation was observed for reaction to YR at the adult plant stage (Figure S1) across both locations.

The estimated variance components for genotype were highly significant (P<0.0001) at both seedling and adult plant stages (Table 1). Similarly, the genotype × environment interaction effect was also highly significant (P<0.0001). Higher disease severity was observed at the Meraro location compared to the Arsi Robe. However, the coefficient of correlation for the infection type to YR between both locations was high (*r* = 0.711). At the seedling stage, 28%, 22% and 50% SHWs exhibited resistant response, intermediate and susceptible responses, respectively. Similarly, 56% and 83% of the SHWs were resistant to YR at the adult plant stage at Meraro and Arsi robe, respectively. Forty-six percent of the SHWs at Arsi Robe and 32% at Meraro scored resistant at the adult plant stages were found to be susceptible to *Pst* at the seedling stage. Twenty-two percent of these were common in both locations (Table S2). A very high level of broad sense heritability (*h^2^_bs_* = 0.88) was observed over the two locations of adult plant YR evaluations.

**Table 1:** Analysis of variance (ANOVA) for reaction to stripe rust in synthetic hexaploid wheat and bread wheat cultivars.

Stage	Source	df	F	Sig
Seedling	Genotypes	186	2.16	***
Adult plant stage	Genotypes (G)	186	3.38	***
	Environments (E)	1	75.64	***
	G×E interaction	186	294.48	***

***Significant difference at P<0.0001.

### Analysis of SNP markers

All wheat lines were genotyped using wheat 8 244 9k Illumina Infinium SNP assay [19]. However, 1 402 (17%) of the SNPs were monomorphic. Three hundred and eighty-eight (4.7%) SNPs were of poor quality and thus were excluded from analysis. The remaining 5 768 (70%) SNPs were further reduced to 2 590 by eliminating markers with minor allele frequency (MAF) ≤0.05. Thus, 2 590 (31.4%) high quality SNPs (Table S5) were used in association analysis for YR resistance using the Q+K MLM method adjusted for false discovery rate *P*<0.05.

A genetic framework map of all 21 wheat chromosomes was constructed using the 2 590 polymorphic SNPs based on the consensus SNP map previously produced by Cavanagh et al. [19] resulting in an average of 123.2 markers per chromosome. However, the marker density for the D genome was relatively poor at 24.14 markers per chromosome. In total, the markers spanned a genetic distance of 3309.5 cM with an average density of 1.27 cM per marker (Table 2). Of the 2 590 SNPs, 2 479 were assigned to 303 unique positions in the wheat genome [19]. The average PIC value for mapped SNPs was 0.25 and 0.30 for unmapped SNPs. A similar trend was observed for diversity (*H*) index.

**Table 2:** Basic statistics of SNP marker polymorphism in synthetic hexaploid wheat.

Chr	*N*	Polymophism	LD	MAF		Diversity (*H*)		PIC	
		(%)	(*r^2^*)	Mean	Range	Mean	Range	Mean	Range
1A	500	93.2	0.2	0.017	0.005–0.49	0.2	0.03–0.55	0.17	0.03–0.45
1B	358	89.11	0.116	0.018	0.005–0.48	0.26	0.03–0.58	0.22	0.03–0.49
1D	95	93.68	0.15	0.018	0.005–0.47	0.27	0.03–0.54	0.23	0.03–0.44
2A	347	90.2	0.157	0.018	0.005–0.49	0.27	0.03–0.56	0.23	0.03–0.46
2B	614	86.97	0.278	0.018	0.005–0.48	0.23	0.03–0.64	0.2	0.03–0.49
2D	100	93	0.086	0.018	0.005–0.48	0.23	0.03–0.53	0.19	0.03–0.43
3A	377	90.72	0.147	0.018	0.005–0.48	0.21	0.03–0.64	0.19	0.03–0.49
3B	421	84.8	0.162	0.018	0.005–0.49	0.21	0.03–0.53	0.18	0.03–0.43
3D	34	89.47	0.178	0.019	0.005–0.49	0.33	0.09–0.54	0.28	0.09–0.44
4A	361	88.37	0.158	0.019	0.005–0.46	0.23	0.03–0.55	0.2	0.03–0.45
4B	168	91.67	0.127	0.019	0.005–0.46	0.28	0.03–0.55	0.24	0.03–0.45
4D	34	67.65	0.63	0.021	0.005–0.14	0.14	0.03–0.51	0.12	0.03–0.45
5A	442	87.78	0.141	0.022	0.005–0.48	0.24	0.03–0.55	0.17	0.03–0.46
5B	540	91.11	0.106	0.022	0.005–0.49	0.2	0.03–0.63	0.17	0.03–0.49
5D	51	84.31	0.2	0.022	0.01–0.39	0.21	0.03–0.53	0.18	0.03–0.42
6A	404	92.08	0.09	0.022	0.005–0.47	0.25	0.03–0.55	0.22	0.03–0.46
6B	410	93.17	0.135	0.022	0.005–0.49	0.24	0.03–0.57	0.21	0.03–0.49
6D	63	87.3	0.104	0.023	0.01–0.44	0.29	0.04–0.60	0.25	0.04–0.49
7A	406	93.6	0.137	0.023	0.005–0.49	0.28	0.03–0.54	0.23	0.03–0.45
7B	284	92.25	0.135	0.023	0.005–0.49	0.25	0.03–0.54	0.22	0.03–0.48
7D	46	82.61	0.088	0.026	0.01–0.48	0.34	0.04–0.63	0.28	0.04–0.49
Unmapped	1130	29.56	-	0.015	0.005–0.32	0.23	0.03–0.66	0.35	0.03–0.49
**Genome**									
A	2837	90.849	0.147	0.02	0.017–0.023	0.24	0.2–0.28	0.201	0.17–0.49
B	2795	89.868	0.151	0.02	0.018–0.023	0.239	0.2–0.28	0.206	0.17–0.49
D	423	85.433	0.205	0.021	0.018–0.026	0.259	0.14–0.34	0.219	0.12–0.49

### Population structure

**Figure 2:**
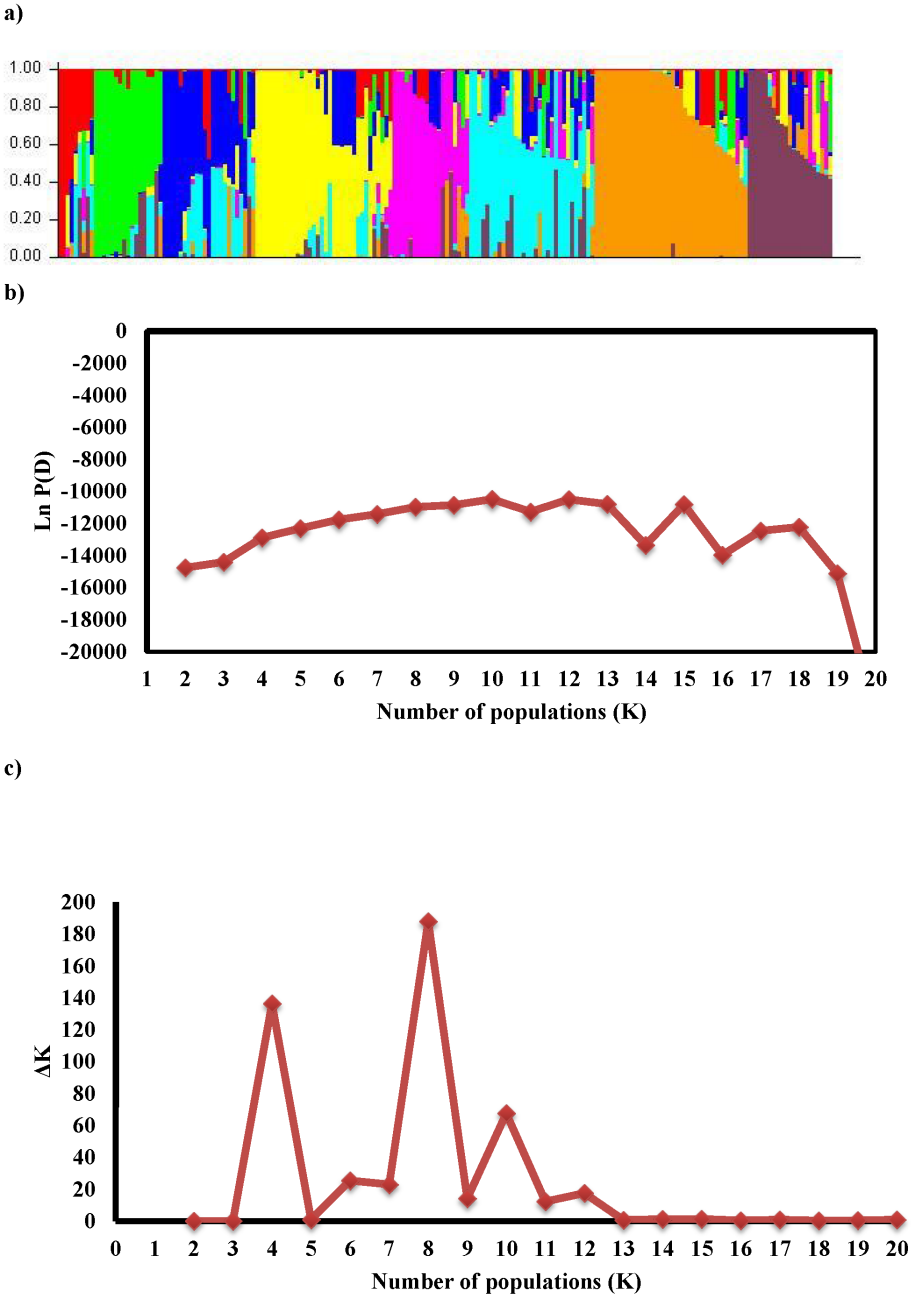
Estimation of number of sub-populations (K) in synthetic hexaploid wheat based on unlinked SNP markers. **a**) Population structure of synthetic hexaploid wheats. The genotype of each line on the figure is represented by a colored line where each color reflects the membership of a cultivar in one of the K clusters. The proportion of the colored segment indicates the proportion of the genome drawn from the K clusters. **b**) The log probability of data as a function of K for unlinked SNP markers. Means log probability of data Ln P (D) for each value of K were calculated from 10 independent runs of structure. **c**) Estimation of number of sub-populations (K) in synthetic hexaploids using deltaK values.

Analysis of population structure showed that the logarithm of the data likelihood (Ln P(D)) on average continued to increase with increasing numbers of assumed subpopulations (K) from 2 to 20 with exception of the depression at K11, K14 and K16 (Figure 2b). However these significant changes at higher K values do not truly reflect the actual number of sub-populations. The ad hoc quantity based on the second order rate of change in the log probability (ΔK) showed a clear peak at K = 8 (Figure 2c), which confirmed that a K value of eight was the most probable prediction for the number of subpopulations. The number of individual SHW lines ranged from 8 in K8 to 50 in K5. The average distance between sub-populations ranged from 0.08 to 0.34, while mean *Fst* value was 0.25.

### Linkage disequilibrium

LD was estimated by *r^2^* at *P*≤0.001 from all pairs of SNPs along each chromosome. On a genome-wide level, 59.68% of all pairs of loci were in significant LD (Table 3) with an average of 0.55. The average *r^2^* of genome-wide LD was 0.19.

**Table 3:** An overview of LD among whole panel of synthetic hexaploid wheat.

Classes	Total pairs	Significant (%)	Significant pairs	Mean r^2^	Pairs in complete LD	Pairs (%) in LD >0.2	Mean of r^2^ >0.2
0–10 cM	33724	77.89	26267	0.44	5732	56.74	0.74
11–20 cM	22913	64.27	14726	0.22	255	34.61	0.55
21–50 cM	48637	57.36	27899	0.14	17	23.60	0.43
>50 cM	77184	51.83	40008	0.1	0	16.02	0.38
Total	182458	59.68	108900	0.19	5644	27.91	0.55

**Figure 3:**
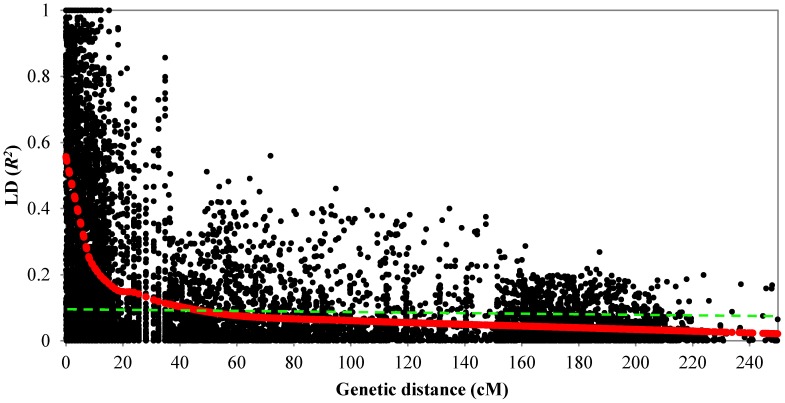
Linkage disequilibrium (LD) decay as a function of genetic distance. *LOESS curve* was fitted by robust locally fitted regression and shows that LD decays with genetic map distance and *dashed line* indicates the derived threshold for LD due to linkage.

SNP markers assigned to their map position were further used to estimate intra-chromosomal LD. About 34.61% of intra-chromosomal pairs of loci were in significant LD with *r^2^*of >0.2 while 5644 SNP pairs were in perfect LD (*r^2^* = 1). The extent and distribution of LD were graphically displayed by plotting intra-chromosomal *r^2^* values for loci in significant LD at *P*≤0.001 against the genetic distance in centi Morgans and a second-degree LOESS curve was fitted (Figure 3). The critical value for significance of *r^2^* was estimated at 0.22 according to Breseghello and Sorrells [33], and thus all values of *r^2^*>0.22 were estimated to be due to genetic linkage. The baseline intersection with the LOESS curve was at 11 cM, which was considered as the estimate of the extent of LD in the SHWs population used for this study, although in a few cases high levels of LD were observed over longer distances (*r^2^* = 1 at a genetic distance of 39.77 cM). LD decays to an average *r^2^* of 0.14 from 0.22 as the genetic distance increased to >10 cM and the markers in complete LD also reduced to 17 from 255 (Table 3). Thus the map coverage of 8–9 cM was deemed appropriate to perform a genome-wide association analysis on the SHWs population since the SNP coverage in this study was at an average density of 1.27 cM per marker.

### Marker-trait associations

**Figure 4:**
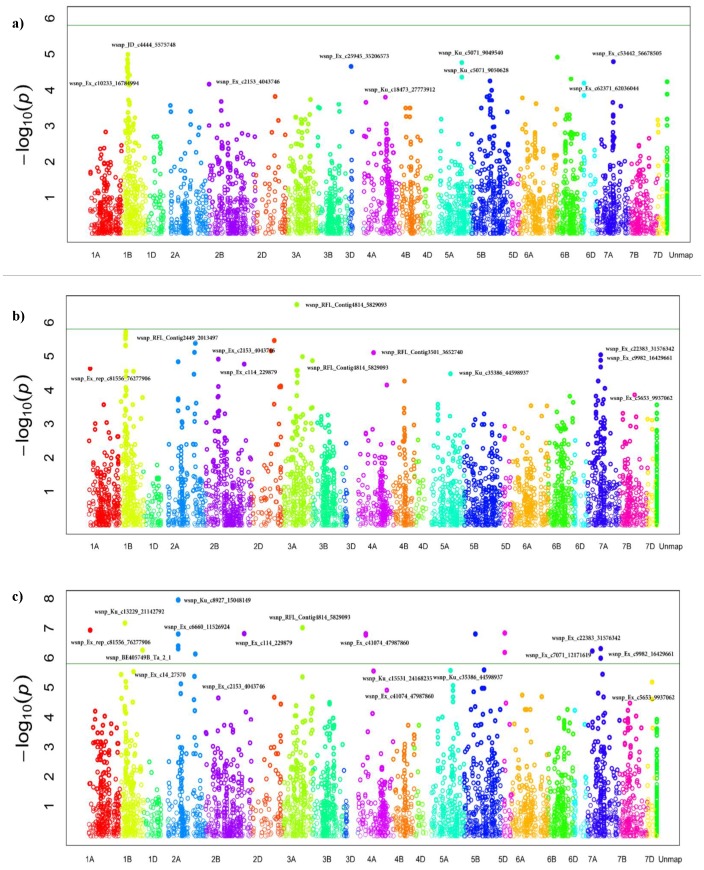
Manhattan plots for statistical significance *P* values across 21 wheat chromosomes for SNP markers associated with stripe rust resistance a) at seedling stage b) At adult plant stage (Meraro) c) Adult plant stage (Arsi Robe). Horizontal line represents the highly significant threshold at *P* 1E-06.

SNP markers associated with resistance to YR at the seedling and adult plant stages were determined by MLM analysis using kinship relationship (K matrix) and population structure (Q matrix) as covariate at FDR-adjusted *P*<0.05. A total of 27 SNPs in nine genomic regions (1BS, 2AS, 2BL, 3BL, 3DL, 5A, 5BL, 6DS and 7A) were identified to be associated with YR resistance at the seedling stage with phenotypic variation (*R^2^*) which ranged from 5.4% to 8.8% (Table 4, Figure 4a). The allele present in cultivar Morocco accounts for susceptibility, therefore, the alternate allele was taken as the resistant allele for YR. The resistance allele frequency (RAF) in SHWs ranged from 9.38 to 93.75%. A haplotype block of 15 SNPs on chromosome 1BS covering a genetic distance from 32 to 40 cM was identified as a major YR resistance gene cluster. Thirteen marker pairs within this block were in perfect LD with an average LD *r^2^* of 0.43. Three more haplotype blocks on chromosomes 2BL, 5AL and 6DS in perfect LD were associated with YR resistance at the seedling stage. The haplotype block of two SNPs (wsnp_Ku_c5071_9049540- and wsnp_Ku_c5071_9050628 (*r^2^* = 1) on 5AL is located at 138 cM. Further, the SNP marker, wsnp_Ex_c53442_56678505 on 7AL was also associated with seedling resistance to YR with *R^2^* and RAF of 6% and 26%, respectively. Since no major YR resistance gene has previously been mapped to chromosome 7A, this may be a novel locus that confers resistance to YR.

**Table 4:** Marker-trait associations for seedling resistance to stripe rust resistance in synthetic hexaploid wheat.

Marker	Chr^a^	Pos^b^	SNP	RAF^c^	MAF^d^	P-value	FDR-adjusted P-value^e^	R^2^	Allelic effect	Gene^f^
wsnp_JD_c4444_5575748	1BS	32	[A/G]	0.94	0.06	3.59E-05	1.42E-02	0.075	0.59	*Yr10* [101], *Yr15* [102], [103], *Yr24* [76], *Yr26* [104], *Yr29* [105], *YrCh42* [79], [80]
wsnp_Ex_rep_c69266_68192766	1BS	34	[T/C]	0.64	0.36	2.71E-05	1.42E-02	0.077	0.59	
wsnp_RFL_Contig2794_2564017	1BS	34	[A/G]	0.89	0.09	2.41E-05	1.42E-02	0.078	0.42	
wsnp_Ex_c11177_18096010	1BS	35	[A/G]	0.64	0.36	3.59E-05	1.42E-02	0.075	0.42	
wsnp_Ex_c14_27570	1BS	35	[A/G]	0.63	0.35	4.01E-05	1.42E-02	0.074	0.39	
wsnp_Ex_rep_c70284_69228305	1BS	35	[T/C]	0.62	0.36	3.15E-05	1.42E-02	0.076	0.42	
wsnp_RFL_Contig2449_2013497	1BS	35	[T/C]	0.90	0.09	2.66E-05	1.42E-02	0.077	0.41	
wsnp_Ku_c62848_63784645	1BS	36	[T/C]	0.62	0.36	5.97E-05	2.00E-02	0.070	0.40	
wsnp_Ex_c38116_45719983	1BS	37	[T/C]	0.59	0.41	3.17E-05	1.42E-02	0.076	0.62	
wsnp_Ku_c30982_40765254	1BS	37	[T/G]	0.89	0.09	3.17E-05	1.42E-02	0.076	0.62	
wsnp_Ku_c30982_40765341	1BS	37	[T/C]	0.51	0.48	1.41E-05	1.42E-02	0.083	0.50	
wsnp_BE442716B_Ta_2_1	1BS	38	[T/G]	0.91	0.08	8.73E-05	2.53E-02	0.067	0.60	
wsnp_Ku_c9014_15193623	1BS	38	[A/G]	0.90	0.08	1.20E-04	2.74E-02	0.064	0.54	
wsnp_Ex_c10233_16784994	1BS	39	[T/C]	0.54	0.45	1.19E-04	2.74E-02	0.064	0.41	
wsnp_Ku_c66585_65967792	1BS	40	[T/C]	0.54	0.44	2.95E-04	4.18E-02	0.057	0.37	
wsnp_Ex_c2772_5130007	2AS	0	[T/C]	0.26	0.26	2.52E-04	4.18E-02	0.058	0.41	*Yr32* [82]
wsnp_Ku_c10418_17237935	2AS	3	[A/G]	0.77	0.19	3.83E-04	4.31E-02	0.055	−0.36	
wsnp_Ex_c2153_4043746	2BL	73	[A/G]	0.88	0.11	4.12E-04	4.38E-02	0.054	−0.73	*Yr5* [103], [106], *YrSp* [84]
wsnp_Ku_c28266_38201643	2BL	73	[A/G]	0.88	0.10	2.58E-04	4.18E-02	0.058	−0.68	
wsnp_Ku_c18473_27773912	3BL	6	[T/C]	0.92	0.07	3.60E-04	4.18E-02	0.055	−0.52	*Yrns-B1* [107]
wsnp_Ex_c25945_35206573	3DL	12	[A/G]	0.51	0.46	2.09E-05	1.42E-02	0.079	0.33	*Yr45* [86]
wsnp_Ku_c5071_9049540	5AL	138	[A/G]	0.09	0.09	7.98E-06	1.42E-02	0.088	−0.53	*Yr34* [108], *Yr48* [78]
wsnp_Ku_c5071_9050628	5AL	138	[A/G]	0.10	0.10	2.03E-05	1.42E-02	0.079	−0.50	
wsnp_Ex_c2582_4804223	5BL	89	[A/G]	0.36	0.36	4.09E-04	4.38E-02	0.054	−0.34	*Yr19* [87]
wsnp_Ex_c62371_62036044	6DS	7	[A/G]	0.71	0.26	1.34E-04	2.74E-02	0.063	−0.28	Novel
wsnp_Ex_rep_c67100_65576598	6DS	7	[A/G]	0.71	0.24	2.54E-04	4.18E-02	0.058	−0.30	
wsnp_Ex_c53442_56678505	7AL	145	[A/G]	0.26	0.26	2.10E-04	3.94E-02	0.060	0.33	Novel

a *Chr* Chromosome,

b *Pos* the marker position on the linkage map (Cavanagh et al 2013),

c *RAF* Resistance allele frequency,

d *MAF* Minro allele frequency,

e False discovery rate (*FDR*) adjusted P values,

f *Gene* the previously reported genes within the same chromosomal regions with reference.

At the adult plant stage, 38 SNPs in 18 different genomic regions were associated with YR resistance at both locations (Table 5, Figure 4b, c). Of these, 15 SNPs in 3 genomic regions (1BS, 3AL, and 5AS) were associated with resistant reaction to *Pst* evaluated at Meraro and 29 SNPs in 18 genomic regions were associated with APR reaction to *Pst* evaluated at Arsi Robe. Six SNPs on chromosomes 1BS, 3AL and 5AS were commonly detected at both locations. The phenotypic variation explained by the SNPs at Meraro ranged from 7.7% for wsnp_Ku_c35386_44598937 on 5AS, to 18.4% for wsnp_Ku_c62848_63784645 on 1BS; while at Arsi robe, *R^2^* ranged from 5.1% for wsnp_Ku_c30982_40765341 on 1BS, to 10% for wsnp_Ku_c8927_15048149 on 2AS.

**Table 5:** Marker-trait associations for adult plant resistance to stripe rust resistance in synthetic hexaploid wheat.

Marker	Chr^a^	Pos^b^	SNP	RAF^c^	MAF^d^	Location	P-value	FDR-adjusted P-value^e^	*R^2^*	Allelic effect	QTLs^f^
wsnp_Ex_rep_c81556_76277906	1AS	120	[T/C]	0.52	0.44	A. Robe	7.22E-04	2.66E-02	0.05	5.60	
wsnp_Ku_c13229_21142792	1BS	8	[T/C]	0.86	0.13	A. Robe	8.81E-05	1.06E-02	0.07	−9.97	
wsnp_BE405749B_Ta_2_1	1BS	10	[T/C]	0.11	0.11	A. Robe	7.17E-04	2.66E-02	0.05	−9.74	
wsnp_CAP7_c2574_1239140	1BS	34	[T/G]	0.71	0.28	Meraro	6.64E-05	3.53E-02	0.105	−4.54	
wsnp_Ex_c3057_5636572	1BS	34	[T/C]	0.74	0.25	Meraro	3.27E-05	1.90E-02	0.114	−4.52	
wsnp_Ex_rep_c69266_68192766	1BS	34	[T/C]	0.63	0.36	Meraro	3.30E-07	4.21E-04	0.176	4.69	
wsnp_Ex_c11177_18096010	1BS	35	[A/G]	0.63	0.36	Meraro	2.86E-07	4.21E-04	0.178	4.73	
wsnp_Ex_c14_27570	1BS	35	[A/G]	0.62	0.35	Meraro	3.03E-07	4.21E-04	0.177	4.43	
wsnp_Ex_rep_c70284_69228305	1BS	35	[T/C]	0.63	0.36	Meraro	2.44E-07	4.21E-04	0.180	4.65	
wsnp_RFL_Contig2449_2013497	1BS	35	[T/C]	0.64	0.35	Meraro	4.70E-07	5.00E-04	0.171	4.58	
wsnp_Ku_c62848_63784645	1BS	36	[T/C]	0.59	0.36	Meraro	1.94E-07	4.21E-04	0.184	4.58	
wsnp_Ku_c30982_40765341	1BS	37	[T/C]	0.48	0.48	Meraro	1.01E-06	9.24E-04	0.161		
						A. Robe	7.72E-04	2.72E-02	0.05	5.21	
wsnp_Ku_c3710_6836438	1BS	37	[T/C]	0.41	0.41	Meraro	1.76E-04	7.48E-02	0.092	3.03	
wsnp_Ex_c10233_16784994	1BS	39	[T/C]	0.45	0.45	Meraro	6.28E-06	5.01E-03	0.136		
						A. Robe	2.35E-04	1.80E-02	0.06	5.44	
wsnp_Ku_c66585_65967792	1BS	40	[T/C]	0.44	0.44	Meraro	1.20E-05	8.48E-03	0.127		
						A. Robe	2.87E-04	1.85E-02	0.06	5.34	
wsnp_Ex_c6660_11526924	2AS	156	[A/G]	0.89	0.10	A. Robe	4.97E-05	7.37E-03	0.08	11.78	QRYr2A.2 [109]
wsnp_Ku_c8927_15048149	2AS	159	[A/C]	0.90	0.09	A. Robe	4.74E-06	3.32E-03	0.10	13.90	QRYr2A.2 [110]
wsnp_be471201A_Ta_1_1	2AS	160	[T/C]	0.89	0.10	A. Robe	2.26E-04	1.80E-02	0.06	11.58	QRYr2A.1 [111]
wsnp_Ex_c1604_3060855	2AS	161	[A/G]	0.90	0.09	A. Robe	4.02E-05	6.25E-03	0.08	12.02	
wsnp_Ex_c2153_4043746	2BL	73	[A/G]	0.88	0.11	A. Robe	1.80E-04	1.70E-02	0.06	−13.04	*Lr23*, QRYr2B.2 [92]
wsnp_Ex_c114_229879	2BS	163	[A/G]	0.87	0.12	A. Robe	1.89E-04	1.70E-02	0.06	−14.22	QRYr2B.4 [92], QYr.ucw-2BS [78]
wsnp_RFL_Contig4814_5829093	3AL	79	[T/C]	0.07	0.07	Meraro	2.19E-05	1.40E-02	0.119	8.57	
						A. Robe	4.12E-04	2.20E-02	0.06	22.35	
wsnp_Ex_c29623_38630871	3BS	102	[A/G]	0.87	0.10	A. Robe	1.37E-04	1.46E-02	0.07	−12.84	QRYr3B.1 [112]
wsnp_Ex_c4267_7700267	3BS	102	[T/C]	0.87	0.10	A. Robe	6.58E-04	2.54E-02	0.05	−11.46	Qyr.ucw-3BS [78]
wsnp_Ex_c4267_7700325	3BS	102	[A/G]	0.88	0.11	A. Robe	1.37E-04	1.46E-02	0.07	−12.59	
wsnp_Ex_c4267_7700461	3BS	103	[T/G]	0.89	0.10	A. Robe	6.58E-04	2.54E-02	0.05	−11.46	
wsnp_BG313770B_Ta_1_1	4AL	57	[A/G]	0.87	0.12	A. Robe	1.72E-04	1.70E-02	0.06	−10.55	QRYr4A.1 [113]
wsnp_Ku_c15531_24168235	4AL	57	[T/C]	0.88	0.11	A. Robe	1.78E-04	1.70E-02	0.06	−11.32	
wsnp_RFL_Contig3501_3652740	4AL	59	[T/G]	0.86	0.14	A. Robe	3.67E-05	5.85E-03	0.08	−10.63	
wsnp_Ex_c41074_47987860	4AL	93	[T/C]	0.91	0.09	A. Robe	9.29E-05	1.10E-02	0.07	10.82	
wsnp_Ku_c35386_44598937	5AS	113	[A/G]	0.88	0.11	Meraro	5.68E-04	9.18E-02	0.077	10.12	
						A. Robe	5.28E-05	7.66E-03	0.07	18.49	QRYr5A.2 [110]; QRYr5A.2 [114]
wsnp_Ex_c807_1586396	5AS	169	[A/G]	0.16	0.16	Meraro	2.83E-04	7.88E-02	0.086	−5.99	
						A. Robe	6.17E-04	2.46E-02	0.05	−14.11	
wsnp_Ra_c5346_9501281	6AS	179	[T/C]	0.08	0.08	A. Robe	4.17E-05	6.33E-03	0.08	−9.76	
wsnp_Ex_c22383_31576342	7AL	41	[T/C]	0.79	0.18	A. Robe	2.00E-04	1.70E-02	0.06	−6.13	QRYr7A.2 [109]
wsnp_Ex_c9982_16429661	7AS	99	[T/C]	0.68	0.31	A. Robe	2.01E-04	1.70E-02	0.06	7.39	QRYr7A.4 [115]
wsnp_Ex_c7071_12171619	7AS	135	[A/G]	0.86	0.13	A. Robe	3.29E-04	2.04E-02	0.06	7.63	QRYr7A.5 [113], [116]
wsnp_RFL_Contig3405_3533915	7BS	65	[T/C]	0.87	0.11	A. Robe	1.04E-04	1.21E-02	0.07	−13.55	QRYr7B.1 [113], [117]
wsnp_Ex_c5653_9937062	7BS	119	[T/G]	0.32	0.32	A. Robe	7.88E-04	2.75E-02	0.05	−5.08	QRYr7B.2 [112]

a *Chr* Chromosome,

b *Pos* the marker position on the linkage map (Cavanagh et al 2013),

c *RAF* Resistance allele frequency,

d *MAF* Minor allele frequency,

e *FDR* False discovery rate adjusted P values,

f *QTLs* the previously reported genes within the same chromosomal regions

**Figure 5:**
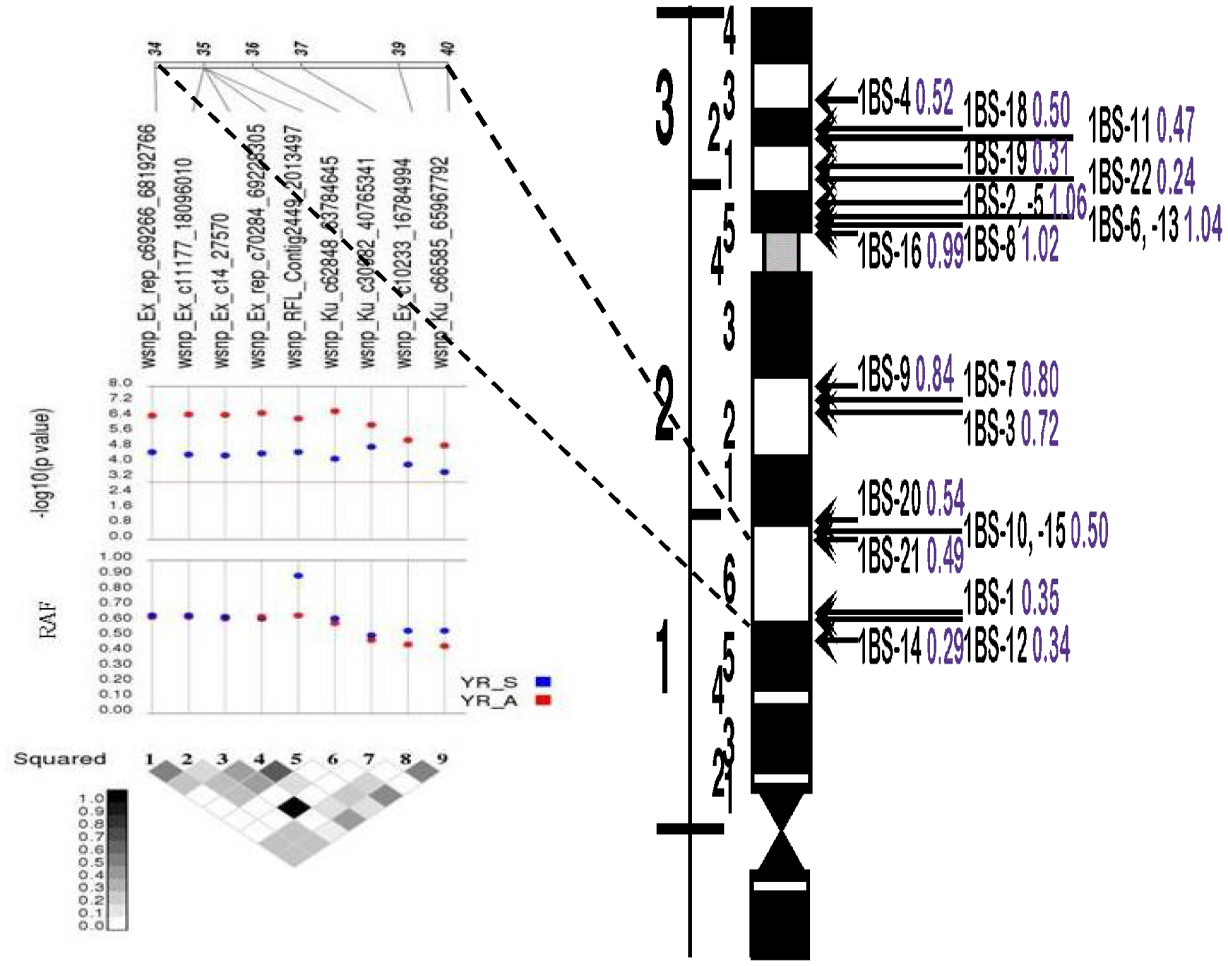
Haplotype block on chromosome 1BS comprising nine SNP markers significantly associated with *Yr* resistance at seedling and adult plant stage (left) aligned with 1BS physical map (right) based on the integrated mapping information (Saintenac et al 2013).

The haplotype block spanning a genetic distance of 34–40 cM on1BS which showed association with YR resistance at the seedling stage, was also associated with adult plant resistance (Figure 5). Similarly, the SNP marker, wsnp_Ex_c2153_4043746, on 2BL was associated with YR resistance at both seedling and adult plant growth stages. The rare allele frequency (RAF) ranged from 8.3% to 90.1%, and in most cases a major allele was associated with resistance in these SHWs, however for 9 SNPs on chromosomes 1BS, 3AL, 5AS, 6AS and 7BS, a minor allele was associated with YR resistance (Table 5). Two additional haplotype blocks associated with YR resistance were identified on chromosomes 3BS and 4AL, each with three SNP pairs that were in perfect LD (*r^2^* = 1). In addition to the major haplotype block on the 1BS chromosome, three other haplotype blocks on 2AS, 3BS and 4AL each with a minimum of two SNPs were associated with YR resistance at the adult plant stage at Arsi Robe.

### *In silico* functional annotation of SNPs associated with resistance to stripe rust

Putative biological functions were determined for SNPs associated with resistance to YR, except for one SNP at chromosome 4AS, two at 2BL and one at 2AS. Owing to the incomplete genome sequencing information of bread wheat, the annotations of the syntenic regions were identified in other cereal crops. These annotations were described for the syntenic regions in DNA and protein sequences of rice, sorghum and *Brachypodium* (Table S3). Putative biological function was redundant for several SNPs within same haplotype block even those with high LD. For example, the closely linked SNPs on chromosome 1BS have similar biological functions.

The emphasis was on the SNPs with important biological functions that have previously been validated and linked with response to disease resistance. The SNPs on 1BS and 7BS were found to correspond to Serine/threonine-protein kinase-like domain disease resistance protein, while the SNPs identified on 5AS, and 7AL corresponded to NBS-LRR proteins, and the SNP on 3AL mapped to an adenosine triphosphate-binding cassette (ABC) transporter family protein. Some regions also encoded transcription factors (TFs) like MADS box TFs (5A) and TFIIE (3DL). One SNP at chromosome 1BS and three at 3BS encoded the important glucosyltransferase family proteins which have been implicated in disease resistance. Similarly, three different SNPs at chromosome 1BS, 2BL and 7BS encoded receptor like kinase proteins which are also an important family of proteins with multiple functions, one of which is disease resistance.

## Discussion

### Phenotypic variability for resistance to stripe rust

One of the main objectives of the current study was to identify SHWs with an adequate spectrum of genetic diversity for resistance against the prevailing YR-virulent races in Ethiopia following the 2010 YR epidemic. Such SHWs could then be crossed with adapted breeding lines to transfer YR resistance into locally adapted wheat cultivars. About 50% of the SHWs in the current study showed intermediate to resistant responses to YR at the seedling stage. Similarly, a high proportion of the SHWs exhibited high level of resistance at adult plant growth stages in Meraro (56%) and Arsi Robe (83%) under field conditions in Ethiopia. Of these, 40 (22%) were common for both locations. The higher disease severity observed at Meraro compared to Arsi Robe may be attributed to environmental effects that affect the incidence and level of disease expressions at different locations. Nevertheless, a high correlation coefficient was observed for YR response data collected between the two locations (*r* = 0.77), thus the adult stage field nursery used in this study provided highly repeatable and reliable phenotypic data for GWAS.

In this study, results from seedling evaluation of SHWs with the Kubsa/Attila isolate that possesses a broad virulence spectrum indicated that 28% of the SHWs were resistant while 22% displayed intermediate resistance while 50% were susceptible indicating the presence of a relatively large number of single major genes in the SHWs which may include uncharacterized major YR resistance genes. Numerous studies have reported that SHWs exhibits natural genetic variation and provide effective sources of resistance against many biotic stresses including fungal and root diseases [7], [8], [9], [38].

The pedigree analysis of SHWs revealed that 26 SHWs developed from durum cultivar, 68.111/RGB-U//WARD RESEL/3/STIL/4, possess better genetic potential to resist *Pst* because none of them exhibited a susceptible response at Meraro while only four displayed an intermediate response at Arsi Robe. Similarly, two SHWs developed from durum cultivar, STY-US/CELTA//PALS/3/SRN-5/4, showed complete resistance to *Pst* at both seedling and adult stages. Ahmed et al. [39] reported the susceptibility of durum varieties Decoy1 and Altar84 to YR at seedling and adult plant stages. In the current study, 25 and 3 SHWs derived from Decoy1 and Altar84 exhibited moderately resistant to resistant response, respectively. Moderately resistant to resistant reaction of the three SHWs derived from Altar84 suggests that the putative source of resistance is likely to be from the *Ae. tauschii* accessions used in producing the SHWs. Similarly, 12 moderate to resistant SHWs derived from Decoy1 possibly acquired their resistance from *Ae. tauschii* accessions. Owing to the absence of disease notes on either the durum or *Ae. tauschii* parents in the current study, it is difficult to pinpoint if the source of resistance is from the durum or *Ae. tauschii* parents in some of the SHWs. For example, *Ae. tauschii* (1016) is the possible source of resistance in SHW117 (DOY1/AE. SQUARROSA (1016)) due to the susceptibility of durum parent (Decoy1), but the same *Ae tauschii* accession in SHW118 (CETA/AE.SQUARROSA (1016)) failed to provide protection to YR. Several SHWs with same durum or *Ae. tauschii* accession displayed variable reaction to YR at both seedling and adult-plant stages. This may be attributed to modulation of gene expression which sometimes occurs when resistance genes are introgressed from species of lower ploidy to higher ploidy. The possibilities of suppression of phenotypic effects in wide crosses of wheat at both interspecific and intergeneric categories have previously been reported [9], [39], [40], [41].

Most importantly in the current study, 22% of SHWs were susceptible at the seedling stage and exhibited moderately resistant to resistant response at adult plant stage at both locations. These accessions may possess adult-plant resistance (APR) against YR. This type of resistance, unlike seedling resistance, is race non-specific and durable [42] and is an important genetic resource for the improvement of wheat against YR. SHWs combine the genomes of tetraploid and diploid wheat progenitors and relatives [6], [7], [9], [43], and they may carry a reservoir of novel genes for resistance to YR. The result obtained in this study lays the foundation for more extensive and in depth studies on the genetic characterization of such potentially novel source of resistance to YR. Another important aspect of the multi-location testing is the identification of loci with broad-spectrum effectiveness across environments. No doubt there are complicated interactions with prevailing environmental conditions including the timing and severity of an epidemic [14]. The chromosomal regions identified in this and earlier studies are likely to contain important loci that are effective across multiple environments.

### Analysis of SNP markers

In this study, only 2,590 of the 8,244 SNPs were selected for genetic diversity, linkage disequilibrium and association mapping studies. The considerable amount of monomorphic SNPs (17%) and SNPs with <0.05 MAF (44.9%) may reflect the different genetic background of the germplasm panel than that of used of SNP marker development. The SNPs present on the array were discovered in a diverse set of 27 wheat lines originating from the US and Australia [19]. The SNP array must therefore be expected to show a higher degree of ascertainment bias when used on SHWs germplasm. However, the quantity of polymorphic SNPs was comparable and more densely saturated compared to the other available marker technologies. For example, Crossa et al. [44] used 813 DArT and 530 SSR and sequence tagged site (STS) markers on 170 lines of CIMMYT wheat germplasm for association mapping (AM) studies, Neumann et al. [45] used 574 DArT markers for AM studies on 96 winter wheat germplasm accessions, Emebiri et al. [46] employed 395 DArT markers for AM studies using 91 SHWs, Rasheed et al. [47] used 895 DArT markers on 231 SHWs, Mulki et al. [48] used 667 DArT markers to identify known and potentially new genomic regions associated with resistance to soil-borne pathogens in SHWs and Tadesse et al. [49] used 1,586 DArT markers to identify MTAs for YR resistance in winter and facultative wheats. Wurschum et al. [50] used the same SNP 9k array to genotype 172 European winter wheat cultivars and found very few SNPs mapped to the D genome in contrast to the results from this study. This may be attributed to the use of SHWs which were derived from a wide array of *Ae. tauschii* accessions which resulted to comparatively more polymorphism compared to conventional bread wheat germplasm. Owing to the comparatively low genetic diversity of the D genome of common wheat [51], [52], the number of markers mapped to the D genome is usually three- to fivefold lower compared to the A and B genomes [19], [53], [54]. The draft genome of *Ae. tauschii* [55], physical map of D-genome [56] and re-sequencing of some SHWs will facilitate the saturation of available SNP assay and future gene discovery studies in SHWs.

### Population structure of synthetic hexaploids

The power of association studies depends on levels of genetic variation, LD and population structure [57]. The existence of population structure can influence association mapping results and lead to false positive trait-marker associations [58], [59]. Identifying and taking into consideration population structure (Q matrix) as a fixed effect and differences in genetic relatedness among lines within the subpopulations (Kinship or K matrix) as random effects reduces the number of false positives [58]. Our results indicated that eight substructures were appropriate in delineating the population structure within the SHWs used in this study. The assignment of the SHWs to the eight subgroups was largely in agreement with their *Ae. tauschii* parent and less so with the durum parent. Two subpopulations were revealed in *Ae. tauschii* [60] using STRUCTURE based on DArT markers. This further validated that the two genetic lineages (L1 and L2) in *Ae. tauschii* accessions have little genetic contact [61]. In SHWs major proportions of genomic diversity are contributed by different accessions of durum varieties and 2 lineages of the D-genome are further influenced by the polymorphisms in A- and B-genomes, resulting in 8 subpopulations. However, the current study analysis carried with STRUCTURE based on only D-genome markers, resulted in only 2 subpopulations (data not shown) consistent with the results of earlier studies [60].

The frequency of *Ae. tauschii* accessions amongst the SHW varied from one to a maximum of four while the durum elite lines ranged from 1 to 26, an indication of the complexity of the crosses. It has been suggested that the STRUCTURE algorithm does not converge to an optimal K when complex genetic structures exist, such as strong relatedness within some germplasm [62]. Our results are within the range of the results obtained by Emebiri et al. [46] who reported that values of K = 8 and/or 9 were sufficient in the grouping of 91 SHW genotypes. Similarly, Mulki et al. [48] reported the value of K = 7 in a collection of 321 SHW genotypes.

### Linkage disequilibrium in synthetic hexaploids

The effectiveness of whole genome association studies for rust resistance as with other traits depends on the decay of LD initially present in a population at a rate determined by the genetic distance between loci and the number of generations since it arose [63]. Numerous studies suggest that LD is not consistent across the whole genome, or along single chromosomes. LD can occur over large distances but may also decrease for nearby loci [45]. Extensive amounts of LD (182 458 SNP pairs) were detected with the 9K wheat SNP platform in this study. A scatter plot of *r^2^* values versus genetic distances between all markers across the genome abruptly declined to 0.2 within 10 cM when all mapped SNPs with chromosome position were analyzed. This result is expected for self-pollinated crop species such as wheat. The estimated genome-wide LD decay in this study ranged from 0–20 cM (Table 3). A very low level of LD (*r^2^* = 0.04) was observed for 6105 SNP pairs.

The quality and estimate LD value is highly conditioned by the distribution of markers and vary according to wheat types and marker systems (e.g., microsatellites and DArT) used. Thus comparison of LD decay values cannot be generalized. LD decay from 10 to 40 cM was detected when advanced breeding lines or wild wheat populations were analyzed by microsatellite and DArT markers [44], [46]. Overall, the extensive LD observed in the present mapping panel corroborates that reported for self-pollinated plants which exhibit considerably high levels of LD. This has been estimated to be about three orders of magnitude higher in wheat, a self-pollinating species, than in maize (*Zea mays* L.), an outcrossing species [33]. The consensus including the results from the present study is that variation in LD patterns in hexaploid wheat are a function of population, genomic region and marker type but generally decay over fairly long genetic distances. Chao et al. [64] suggested that observed divergence in the extent of LD between eight subpopulations of spring wheat was attributed to unique breeding histories and selection pressures targeted to genes located in the different genomes during the process of cultivar development. The SHWs and their derivatives have increased genetic diversity than the hexaploid wheat, particularly in the D-genome [65]. A similar case is observed in these SHWs where unusual patterns of LD, rate of LD decay and lower pairwise *r^2^* values are attributed to the genomic constitution of the germplasm. It is well known that the introduction of new haplotypes from divergent population can increase the extent of LD [57].

### Alignment of QTLs to previously identified YR genes/QTLs

This study identified nine and eighteen different genomic regions associated with YR resistance at the seedling and adult plant stages, respectively using MLM corrected for population structure and familial relatedness adjusted for false discovery rate at *P*-values of ≤0.005 or ≤0.05. To date, 84 YR resistance genes have been designated in wheat out of which 36 have temporary designations [3]. There are several YR resistance genes derived from wild relatives including *Yr5* from *T. spelta* [66], *Yr8* from *Ae. comosa* [67], *Yr9* from *Secale cereale* [68], *Yr28* from *Ae. tauschii* [69], *Yr37* from *Ae. kotschyi* [70], *Yr38* from *Ae. sharonensis* [71], *Yr40* from *Ae. geniculata* [72] and *Yr42* from *Ae. neglecta* [73]. Several studies have identified and in some cases mapped YR resistance genes in SHWs [8], [74], [75], [76], [77], [78]. The YR resistance gene, *Yr28*, on chromosome 4DS was derived from a synthetic hexaploid [69]. Another YR resistance gene, *YrCH42*, was derived from Chinese wheat cultivar ‘Chuanmai 42’ which was a synthetic derived germplasm from CIMMYT wheat program. *YrCh42* mapped to 1BS and was reported as allelic with *Yr24* and *Yr26* [79] and they were later shown to be the same gene [80]. Recently, Lowe et al. [78] reported the mapping and validation of YR resistance gene, *Yr48* on chromosome 5AL, that confers partial resistance to broadly virulent post-2000 North American races of YR in wheat which is derived from SHWs. Similarly, Bux et al. [81] postulated the likely presence of YR seedling resistance genes; *Yr3, Yr5, Yr10, Yr15, YrSP* and *YrCV* amongst 95 SHWs evaluated for seedling resistance and under field conditions at two hot spot locations in Pakistan.

The current study identified a major haplotype block on chromosome 1BS with 15 SNPs associated with resistance to YR at seedling stage. Nine SNPs within this haplotype block were also associated with YR resistance at adult plant stage suggesting the possible presence of more than one gene/locus conferring resistance to YR within this genomic region. Previously, seven major YR resistance genes (*Yr10, Yr15, Yr24/Yr26/YrCH42, YrH52*, and *YrC142*) have been mapped to chromosome 1BS out of which 3 are temporarily designated [3]. Wen et al. [80] previously mapped *Yr24/Yr26/YrCh42* to chromosome 1BS and its source was a Chinese wheat cultivar Chuanmai42 which is a D genome synthetic derivative [79]. It is most likely that the region on 1BS identified in the current study may be *Yr24/Yr26/YrCh42* [80] or an allelic form of this combination. However, there is also the possibility of other genes or QTLs within proximity of this haplotype block.

Another haplotype of two SNPs on chromosome 2AS associated with *Pst* was identified in the proximity of the previously mapped gene, *Yr32* [82]. This gene is still effective and confers resistance to YR isolates collected in Kenya [83]. *Yr1* is also located on chromosome 2AS but the virulence analysis of *Pst* isolates suggested that these genes are no longer effective against YR [83]. Similarly, a haplotype block with two SNPs on chromosome 2BL identified in the current study may be within proximity of the *Yr5* gene. *Yr5* is one of major genes for which no virulent *Pst* isolate has been found worldwide [83] and is effective against all races of *Pst*. This gene is allelic with *Yr7* and *YrSp* (Spalding Prolific) [84]. *Yr7* is ineffective against the *Yr27* virulent race but *YrSp* is resistant against this race [85]. Given that the breakdown of resistance to *Yr27* is the major cause of near epidemic proportions of YR experienced in Ethiopia in 2010, it is likely that the markers identified in the current study on 2BL may be associated with the resistance conferred by *Yr5* and/or *YrSp*. This needs to be furthered investigated.

Two D-genome encoded regions associated with YR are important because they might have novel alleles due to the use of untapped *Ae. tauschii* accessions. It is most likely that a single SNP on chromosome 3DL associated with reaction to *Pst* identified in the current study is linked to *Yr45*. *Yr45* is the only gene reported on 3DL [86], and integrated genetic map information strongly suggests that *Yr45*-linked SSR markers close to the SNP identified in this study are linked to YR resistance on 3DL [30].

*Yr34* and *Yr48* are major *Yr* genes mapped to chromosome 5AL where two SNPs were associated with resistance to YR in the current study. No information is available in the literature about the virulence in *Pst* to these genes. However, we were unable to align these SNPs with either of the genes using the integrated genetic map [30]. It is most likely that this region may be *Yr48* because this gene is derived from synthetic hexaploids [78]. The current study identified an SNP on chromosome 5BL linked with seedling resistance to YR, which appears to co-locate with genomic regions of catalogued YR resistance genes. Genes mapped to chromosome 5BL include *Yr19* [87], *YrDu* [88] and *YrExp2* [89]. Sharma-Poudyal et al. [83] reported that all *Pst* isolates from Kenya were found to be virulent to *YrExp2*.

MacGene catalogued more than 100 QTLs conferring resistance to YR in bread and durum wheats [3]. Rosewarne et al. [14] surveyed QTLs for YR resistance and reported that 140 QTLs on 49 genomic regions have been identified in various studies. Earlier, Crossa et al. [44] used GWAS to map rust resistance loci in 170 wheat lines with 813 DArT markers. They identified a total of 275 DArTs (63 for stem rust, 90 for leaf rust and 122 for yellow rust) to be associated with disease resistance which were spread across all wheat chromosomes except 6D. The positions of SNP markers declared significant in the present study were compared to the YR QTLs reported in wheat consensus maps (http://ccg.murdoch.edu.au/cmap/ccg-live/). Thirteen out of 18 genomic regions co-locate with known genes and/or QTL for YR resistances (Table 5). The alignment of QTL with catalogued YR resistance genes further validates the accuracy of the association mapping approach used in the current study. The chromosomal regions identified in numerous studies are likely to contain important loci that are effective across multiple environments and may warrant a greater focus for future research. QRYr2A.1 was identified in seven studies and appeared to be a gene rich region containing several seedling resistances and, as well as some minor QTLs. QRYr2A.1 also corresponds to a region with several translocations. The pleiotropic adult plant resistance (PAPR) feature is an important component of resistance to biotrophic fungi. An important region identified to be associated with YR is 5AL (138 cM) which has been associated with both stem and YR resistance [90], suggestive of pleiotropic gene effects. Similarly, QTL on 2BS in this study can be aligned to QYrlo.wpg-2BS [91] and QYr.ucw-2BS [78], the latter being derived from SHWs and encode partial resistance to a mixture of broadly virulent *Pst* races. Both reported QTLs are strongly linked to the same SSR marker (wmc474) which may indicate that they may be allelic. Additionally, another QTL, QRYr2B.4 [92] is likely to be the same region based on the consensus Yr QTL mapping information [14]. The consistent detection of this QTL across diverse environments is an indication of its versatility in providing resistance to not only the post 2010 *Pst* races but of its potential utility in transferring resistance to YR susceptible cultivars in Ethiopia.

### New loci for YR resistance

A single SNP on chromosome 7AL at the 145 cM position is most probably a new gene as there is no major YR gene in literature present on chromosome 7AL. The resistance allele for this SNP is present in 26.4% SHWs. Similarly, the SNP identified on chromosome 6DS may be new since the previously mapped genes viz. *Yr20, Yr23*, *YrTy2* and *YrTr1* [87], on this chromosome are virulent to *Pst* isolates from Kenya [83]. This warrants further investigation and confirmation through bi-parental mapping. No APR QTL is reported within proximity of the 1BS haplotype block consisting of 12 SNPs and its association with APR may be due to some major gene/s or a new QTL. Similarly, the remaining four regions (1AS-12cM; 1BS-8,10 cM; 3AL-70 cM; 6AS-179 cM) were not aligned to any previously published QTLs, hence these are most likely novel QTLs. These QTLs are on located on the A and B genomes contributed by the durum parents of SHWs and can be important sources to introduce novel durum genomic diversity to bread wheat.

### *In silico* analysis of SNPs associated with stripe rust resistance

The exponential availability of sequence information for crops species is facilitating more effective study of target loci underlying important traits. In the current study, putative biological functions were identified for 46 SNPs associated with YR resistance (Table S3). The gene and protein families include *R* (resistance) superfamily, encoding nucleotide binding site leucine-rich repeats (NBS-LRR) domains flanked by the two SNPs, wsnp_Ex_c807_1586396-5AS and wsnp_Ex_c22383_31576342-7AL. The SNP on 5AS is noteworthy since it is associated with YR resistance at the adult plant stage across both locations. Similarly, an ABC transporter is another important gene family associated with the SNP, wsnp_RFL_Contig4814_5829093, on 3AL which conferred YR resistance at adult plant stage across both locations. The ABC transporter gene family is known to confer durable resistance to multiple fungal pathogens in wheat [93]. The other important gene families include protein kinase (wsnp_Ex_c7071_12171619-7AS; wsnp_Ex_c5653_9937062-7BS; wsnp_Ku_c13229_21142792-1BS; wsnp_Ex_c2153_4043746-2BL; wsnp_Ku_c28266_38201643-2BL), transporters (wsnp_RFL_Contig2794_2564017-1BS; wsnp_Ex_c38116_45719983-1BS; wsnp_Ex_c6660_11526924-2AS; wsnp_Ku_c8927_15048149-2AS; wsnp_Ex_c4267_7700267-3BS) and proteases (wsnp_Ex_c29623_38630871-3BS; wsnp_RFL_Contig3501_3652740-4AL). Previously, Marone et al. [94] conducted a wide survey by *in silico* analysis of sequences of wheat specific DArT markers and identified the same gene families frequently associated with DArT loci known to be associated with disease resistance. Similarly, Marone et al. [95] identified the receptor like protein kinase corresponding to powdery mildew resistance in wheat. Joukhadar et al. [96] identified DArT loci associated with pest resistance encoding Ser-Thr kinase domain. Qamar et al. [97] reported that the tomato resistance gene *TPK1b* is required for resistance to insect feeding and this gene contains the Ser-Thr kinase domain. These proteins are likely involved in lipid metabolism, amino-acid biosynthesis or cell wall modifications upon the attack of rust pathogen. In particular, receptor like kinase proteins (7AS, 7BS, 1AL, and 1BS) are involved in cell wall modifications, NBS-LRR proteins (5AS and 7AL) are involved in ‘secretion and transportation’, and transcription factors (3AL) and transporter proteins (1BS, 2AS and 3BS) are involved in amino acid and lipid metabolisms.

Recently, Fu et al. [98] characterized actin-depolymerizing factor, *TaADF7*, family which was mapped on group 1 chromosomes and regulates actin cytoskeleton dynamics and encodes hypersensitive response against wheat stripe rust. The 1BS encoded copy of *TaADF7* was localized to wheat contig IWGSC_3442815 through *in silico* approach, while several SNPs on1BS (32–35 cM) were developed within this contig. This strongly suggests the function of potential candidate gene within this region may be actin-depolymerizing factor. The successful detection of this region by forward GWAS could further be validated by reverse genetic approach to identify specific genes involved.

### Further insight into *TaAbc1* and *TaLSD1* genes encoding hypersensitive resistance to stripe rust and possible exploitation in SHWs

Plant hypersensitivity response (HR) as function of rapid cell death at the site of infection, is regarded as one of the most efficient defense response to microbial pathogens. Recently, Wang et al. [99] reported the cloning of *TaAbc1* gene from the wheat cultivar Suwon11. This gene is reportedly a rust-pathotype specific HR mediator and is only triggered by avirulent YR pathotypes. The demonstrated ability of this gene to encode YR resistance provides opportunities to search for its homoeologues in different wheat genomes. The recent availability and access to relatively complete genome sequence of bread wheat (wheat genome survey sequence), and draft genome sequence of *Ae. tauschii* [55] will allow more in-depth and comprehensive studies for putative wheat homoeologs of large gene families. In the current study, the SNP wsnp_RFL_Contig4814_5829093 on 3AL encode ABC transporter protein, however its sequence homology with *TaAbc1* genes was very low (>50% with E = 3e-40) which diminishes the likely of this region as candidate *TaAbc1* homolog. However, the sequence of conserved ABC1 protein family domain was used to query and blast the wheat genome survey sequence database (http://urgi.versailles.inra.fr/Species/Wheat/Sequence-Repository, accessed 10 March 2014). A significant hit was found on chromosome 5DL (overall 75% similarity, E = 1e-21) making this region an important candidate to explore and functionally validate the effect of this gene on YR resistance. SHWs and their derived advanced lines may offer required variability to validate the role of this gene compared to conventional bread wheats.

Similarly, *TaLSD1* is another recently cloned gene in wheat [100] which is a lesion stimulating disease 1 (lsd1) gene that belongs to zinc finger subfamily that exhibits a runaway cell death phenotype once initiated by superoxidase-dependent signals. In the current study, flanking sequence of SNP, wsnp_Ex_c2582_4804223-5BL, associated with seedling *Pst* resistance had synteny to zinc finger domain in *Brachypodium* (Table S3), however, this domain is different to zf-LSD1 domain. The complete coding sequence of *TaLSD1* was used as a query and blasted to survey sequence database and a significant hit was found on chromosome 1DL (scaffold 2251473) with high similarity (93%, 540bp, E = 4e-148) (Figure S2). The pfam annotation of this scaffold region transcribed into zf-LSD1.3 (ID: PF06943). This significant higher identity makes this region important candidate for validation and subsequent functional analysis of *TaLSD1* gene family members in *Ae. tauschii* through characterizing SHWs.

The QTLs and SNPs identified in the current study will be of considerable interest to the wheat community. However, it is, essential to validate these QTLs by using bi-parental populations or near-isogenic lines (NILs) and testing their utility across multiple environments.

## Supporting Information

Figure S1 Frequency distribution of synthetic hexaploids evaluated to stripe rust at seedling stage (a) and adult plant stage at Meraro (b) and Arsi Robe (c).
(DOCX)

Figure S2 Alignment of hypersensitive response (HR) mediated programmed cell death (PCD) encoding genes involved in stripe rust resistance, with sequences from wheat D-genome. (**a**) Alignment of *TaAbc1* gene with highest similar sequence from chromosome 5DL contig 4570425. (**b**) Alignment of *TaLSD1* gene with highest similar sequence from chromosome 1DL scaffold 2251473.
(DOCX)

Table S1 Pedigree information of synthetic hexaploids and bread wheat cultivars used to evaluate resistance to stripe rust.
(XLSX)

Table S2 List of synthetic hexaploids putatively possessing adult plant resistance (APR) to stripe rust.
(XLSX)

Table S3 *In silico* functional analysis of SNPs associated with stripe rust resistance in synthetic hexaploids.
(XLSX)

Table S4 Stripe rust terminal disease scores, AUDPC of SHWs at Meraro and Arsi Robe and seedling test in green house during 2011.
(XLS)

Table S5 High quality SNPs used for GWAS for stripe rust resistance in synthetic hexaploid wheats.
(XLSX)
