# Supplementary material for: Genome-Wide Association Mapping for Seedling and Adult Plant Resistance to Stripe Rust in Synthetic Hexaploid Wheat
Source: PLoS One. 2014 Aug 25;9(8):e105593. doi: 10.1371/journal.pone.0105593 (PMC4143293; doi:10.1371/journal.pone.0105593)
Supplement: Figure S1 — Frequency distribution of synthetic hexaploids evaluated to stripe rust at seedling stage (a) and adult plant stage at Meraro (b) and Arsi Robe (c). (DOCX) [file pone.0105593.s001.docx]

a)


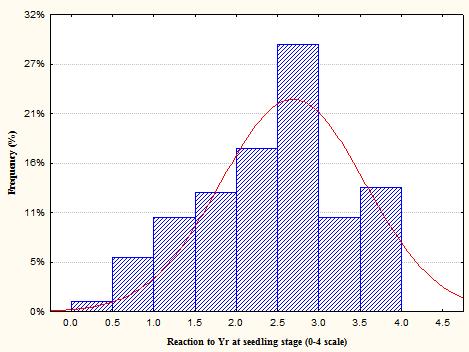


b)


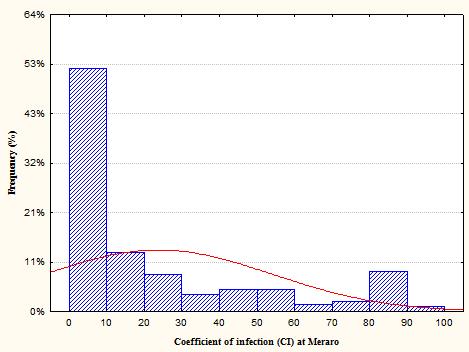


c)


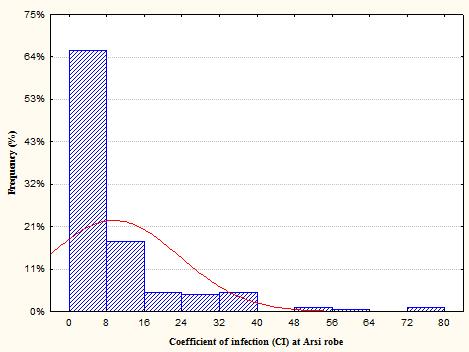


**Figure S1**: Frequency distribution of synthetic hexaploids evaluated to stripe rust at seedling stage (a) and adult plant stage at Meraro (b) and Arsi Robe (c)
