# Supplementary material for: Genome-Wide Association Mapping for Seedling and Adult Plant Resistance to Stripe Rust in Synthetic Hexaploid Wheat
Source: PLoS One. 2014 Aug 25;9(8):e105593. doi: 10.1371/journal.pone.0105593 (PMC4143293; doi:10.1371/journal.pone.0105593)
Supplement: Figure S2 — Alignment of hypersensitive response (HR) mediated programmed cell death (PCD) encoding genes involved in stripe rust resistance, with sequences from wheat D-genome. (a) Alignment of TaAbc1 gene with highest similar sequence from chromosome 5DL contig 4570425. (b) Alignment of TaLSD1 gene with highest similar sequence from chromosome 1DL scaffold 2251473. (DOCX) [file pone.0105593.s002.docx]

**a)**


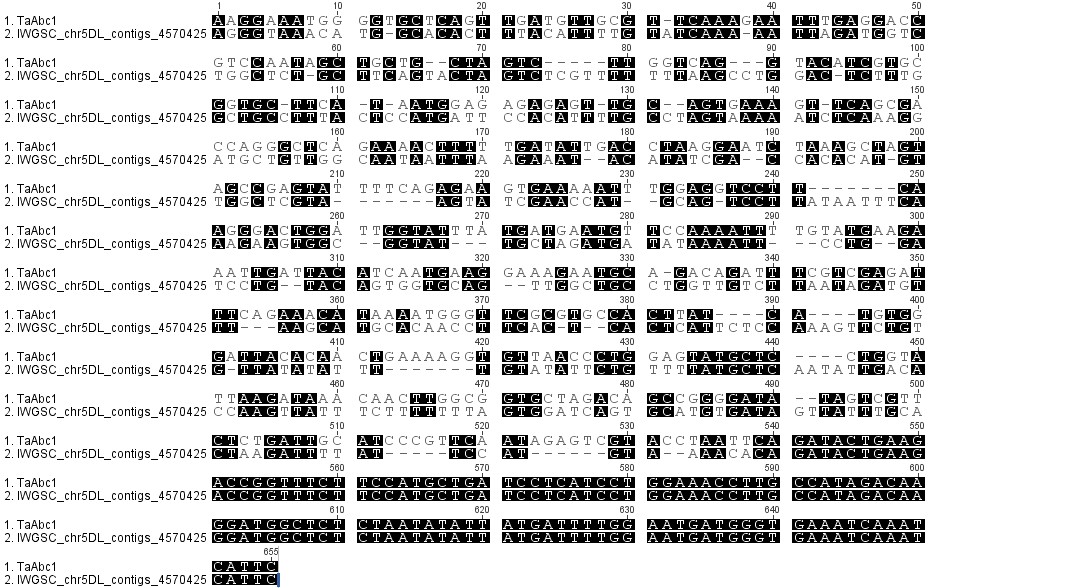


**b)**


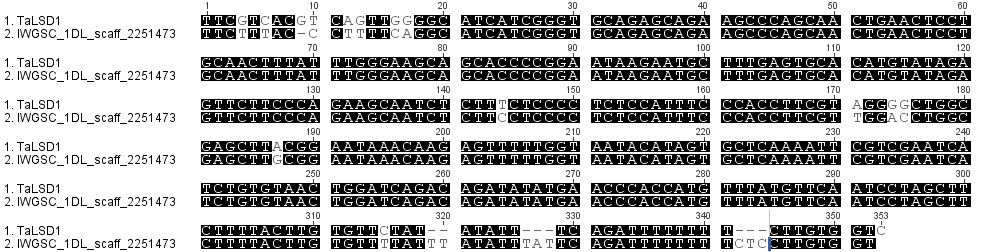


**Figure S2:** Alignment of hypersensitive response (HR) mediated programmed cell death (PCD) encoding genes involved in stripe rust resistance, with sequences from wheat D-genome. **(a)** Alignment of *TaAbc1* gene with highest similar sequence from chromosome 5DL contig 4570425. **(b)** Alignment of *TaLSD1* gene with highest similar sequence from chromosome 1DL scaffold 2251473.
